# Supplementary material for: The Construction and Exploration of a Comprehensive MicroRNA Centered Regulatory Network in Foxtail Millet (Setaria italica L.)
Source: Front Plant Sci. 2022 May 6;13:848474. doi: 10.3389/fpls.2022.848474 (PMC9121102; doi:10.3389/fpls.2022.848474)
Supplement: Supplementary file 4 [file Data_Sheet_4.zip › Sit-miR398b_Seita.7G206200.1_683_TPlot.pdf]

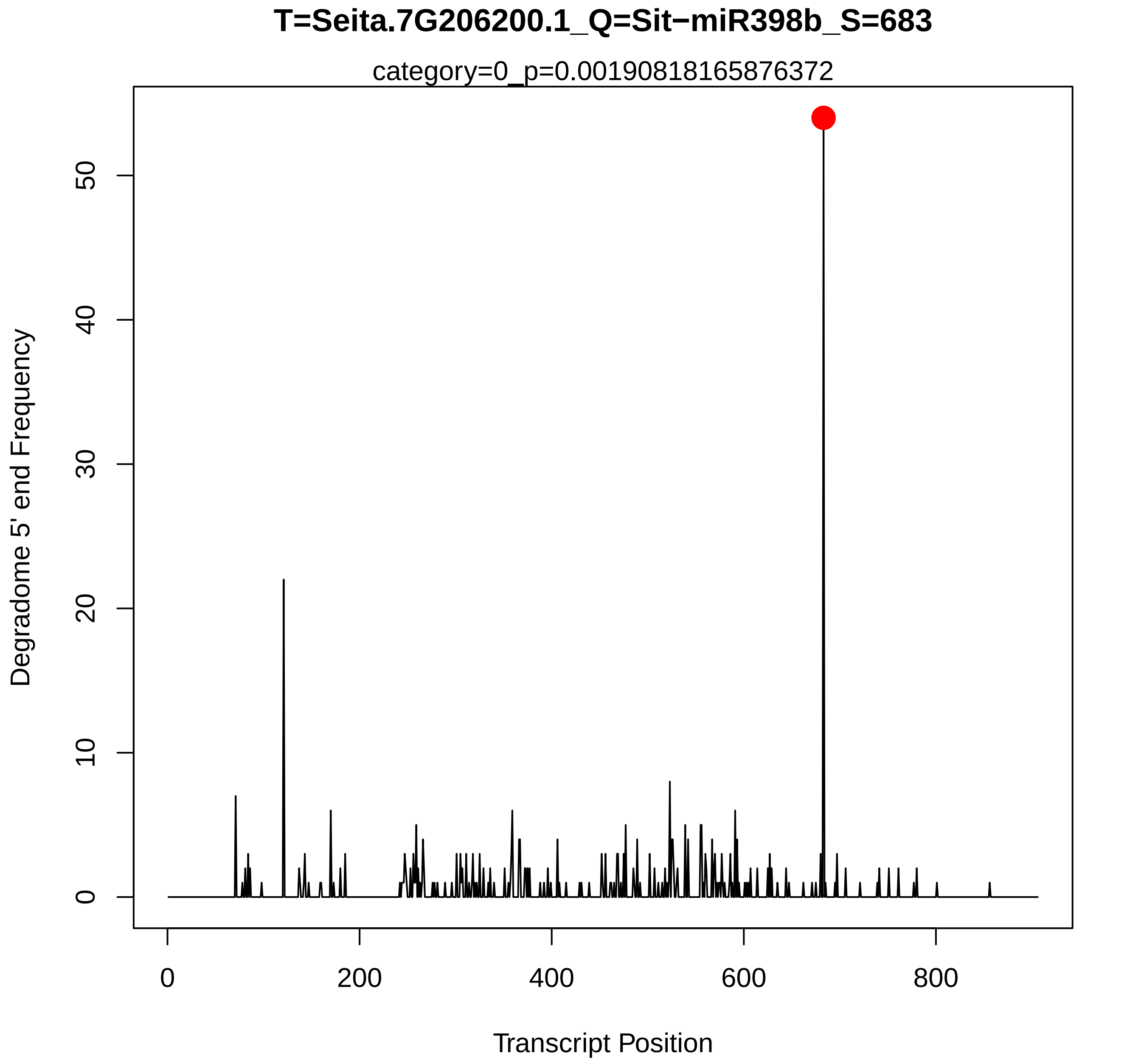
T=Seita.7G206200.1_Q=Sit−miR398b_S=683
category=0_p=0.00190818165876372
Degradome 5' end Frequency
0
10
20
30
40
50
0
200
400
600
800
Transcript Position
